# Genome-wide analysis of *PIN* genes in cultivated peanuts (*Arachis hypogaea* L.): identification, subcellular localization, evolution, and expression patterns

**DOI:** 10.1186/s12864-023-09723-5

**Published:** 2023-10-21

**Authors:** Jianxin Bian, Yuanyuan Cui, Jihua Li, Yu Guan, Shuhua Tian, Xiaoqin Liu

**Affiliations:** Peking University Institute of Advanced Agricultural Sciences, Shandong Laboratory of Advanced Agricultural Sciences in Weifang, Weifang, Shandong 261325 China

**Keywords:** Peanut, PINs, Genome-wide identification, Subcellular localization, Expression patterns

## Abstract

**Background:**

Auxin is an important hormone in plants and the PIN-FORMED (PIN) genes are essential to auxin distribution in growth and developmental processes of plants. Peanut is an influential cash crop, but research into *PIN* genes in peanuts remains limited.

**Results:**

In this study, 16 *PIN* genes were identified in the genome of cultivated peanut, resolving into four subfamilies. All *PIN* genes were predicted to be located in the plasma membrane and a subcellular location experiment confirmed this prediction for eight of them. The gene structure, cis-elements in the promoter, and evolutionary relationships were elucidated, facilitating our understanding of peanut *PIN*s and their evolution. In addition, the expression patterns of these *PIN*s in various tissues were analyzed according to a previously published transcriptome dataset and qRT-PCR, which gave us a clear understanding of the temporal and spatial expression of *PIN* genes in different growth stages and different tissues. The expression trend of homologous genes was similar. *AhPIN2A* and *AhPIN2B* exhibited predominant expression in roots. AhPIN1*A-1* and *AhPIN1B-1* displayed significant upregulation following peg penetration, suggesting a potential close association with peanut pod development. Furthermore, we presented the gene network and gene ontology enrichment of these PINs. Notably, AhABCB19 exhibited a co-expression relationship with AhPIN1A and AhPIN1B-1, with all three genes displaying higher expression levels in peanut pegs and pods. These findings reinforce their potential role in peanut pod development.

**Conclusions:**

This study details a comprehensive analysis of *PIN* genes in cultivated peanuts and lays the foundation for subsequent studies of peanut gene function and phenotype.

**Supplementary Information:**

The online version contains supplementary material available at 10.1186/s12864-023-09723-5.

## Background

Plant growth and development are regulated by a variety of plant hormones, among which auxin was the first discovered to participate throughout the plant life cycle, making it indispensable [1]. Auxin is involved in many biological processes, such as embryogenesis, cell division, root growth, phototropism, and geotropism [2]. Auxin is predominantly synthesized in the apical meristem and moves among cells through a combination of membrane diffusion and carrier-mediated transport [3]. Membrane diffusion depends on the concentration gradient of auxin and requires no energy consumption and has no fixed direction. Carrier-mediated transport is directional flow that is critical to plant morphogenesis and adaptation [4]. Studies about Arabidopsis show two types of auxin carriers: influx and efflux. The former includes AUX1 (AUXIN-RESISTANT 1) and the LAX (LIKE AUXIN) family and the latter includes the PIN (PIN-FORMED) and PGP/ABCB (P-glycoprotein/ATP-binding cassette-B) families [5]. The polar location of PIN protein is closely related to the direction of auxin flow between cells, which is dynamic and strictly controlled on certain sides of specific cells, regulating important growth and developmental processes in higher plants [6].

Tropism is an adaptive response of plants to environmental stimuli, and polar auxin movement is the main cause of organ curvature due to cell growth differences in most tropism responses [7]. For example, the downward growth of plant roots caused by gravity is mainly due to the polar distribution of auxin [8]. In Arabidopsis, many studies support an essential role of PIN-mediated directed auxin flow in the root gravity response [9]. There are eight PIN members in Arabidopsis and PIN2, PIN3, and PIN7 are all involved in root gravitropism [10]. AtPIN2 plays a critical role in mediating auxin transport in root gravitropism, which transmits gravity signals perceived in the root cap to the root elongation zone [11]. The loss of function *PIN2* mutant exhibits a gravity-insensitive root growth phenotype [12]. After gravity stimulation, the abundance of PIN2 decreases in the upper root epidermis, and increases in the lower root epidermis. The asymmetric abundance of PIN2 enhances the transport of polar auxin to the root extension zone, resulting in downward root growth [13]. Moreover, PIN3 and PIN7 also change their positions after gravity stimulation through endocytosis. By default, both PIN3 and PIN7 are located on the plasma membrane of columnar cells. After gravity stimulation, they rapidly move to the new lower sides of columella cells, driving auxin flow towards the lower side of the root tip [14].

Peanut is a geocarpic species that produces pods within the soil. Its special organ, the gynophore or peg, is an elongated pistil stalk with root-like gravitropism, pushing the ovary into the soil and forming the pod [15]. Roots of higher plants exhibit gravitropism, while shoots exhibit negative gravitropism [16]. Although the gynophore is attached to the aboveground part of the peanut plant, it is gravitropic like roots rather than negatively gravitropic like shoots [17]. Immunolocalization experiments showed that IAA (indoleacetic acid) was distributed in the intercalary meristem, epidermis, and cortex of the elongation region and the region near the seed of the vertically growing gynophores [18]. IAA redistributes to the upper side of the epidermis and cortical regions of peanut gynophores following horizontal reorientation, after which the growth rate of the upper side of the gynophores was faster than that of the lower side, promoting downward growth [18]. These results suggest that the asymmetric distribution of auxin is also the basis of the gravitropic response of peanut gynophores.

Interestingly, in both roots and stems, auxin responds to gravity by accumulating in the lower side of the tissue, whereas in gynophores, it accumulates in the upper side in response to gravity. In addition, although gynophores exhibit gravitropism like roots, their response to auxin is consistent with that of shoots (auxin promotes cell expansion) rather than roots (auxin inhibits cell expansion). This indicates that the gynophore is a special organ, with traits like both shoots and roots. Transcriptomics and proteomics have also been used to explore genetic and protein changes in peanut gynophores in response to gravity. Auxin related genes and proteins have been respectively identified as differentially expressed or abundant, such as auxin conjugate hydrolase, auxin efflux carrier, and ABC transporter [19–21]. However, further research on these genes and proteins is scarce.

In this study, a comprehensive analysis including identification, subcellular locations, gene structure, cis-elements, evolutionary relationships, transcriptional expression, and interaction networks of *PIN*s in cultivated peanut was performed. The goal was to give researchers a basic understanding of these genes in peanut to facilitate subsequent research.

## Results

### Identification and phylogenetic analysis of *PIN* genes in peanut

**Fig. 1:**
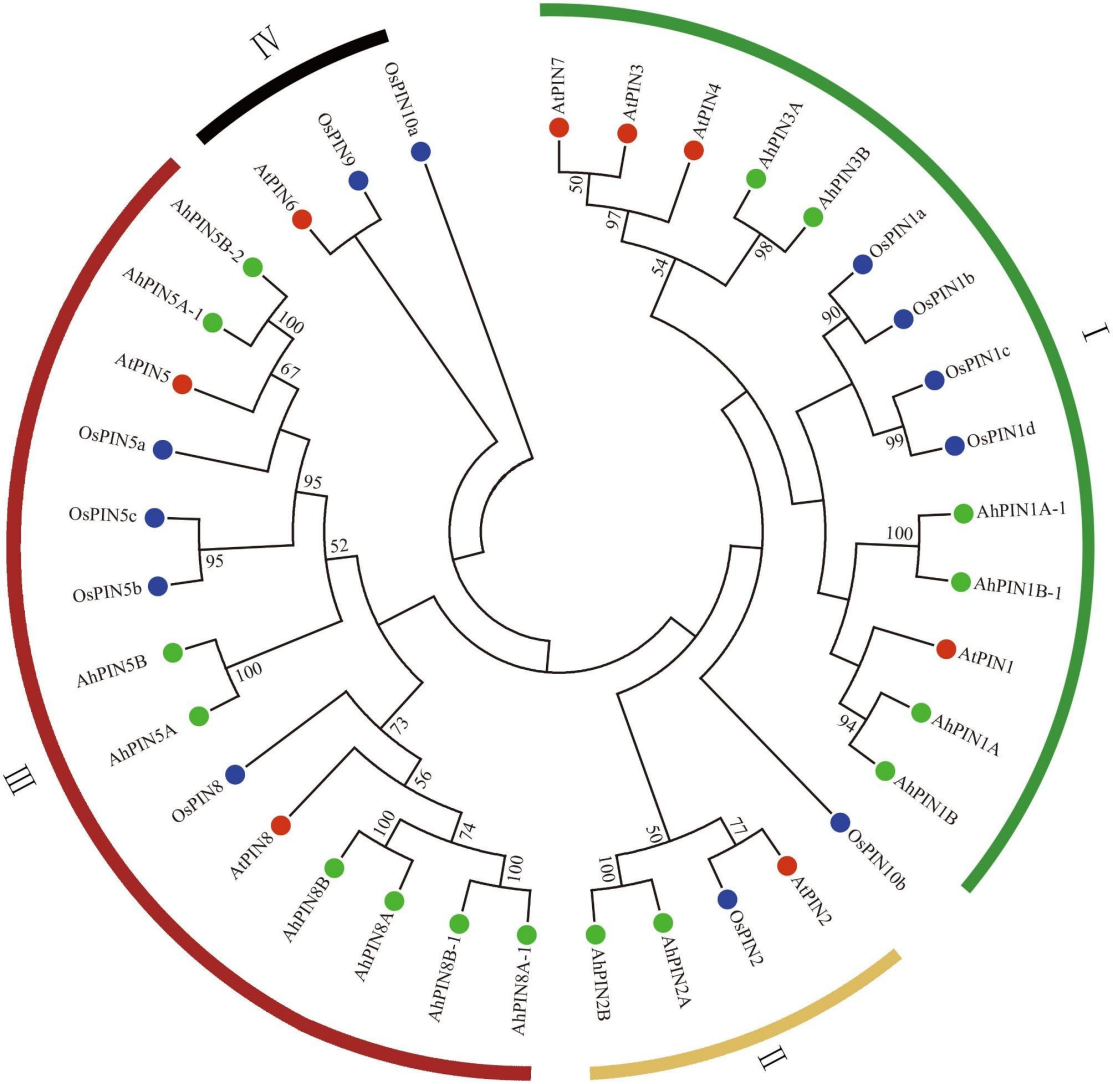
Evolutionary tree of AtPIN, OsPINs and AhPINs. The details of these genes could be found in Supplemental Table 1

A total of 27 *AhPIN* genes were initially identified in the peanut genome (Supplemental Table 1). However, subsequent to a comprehensive motif analysis, we excluded 11 *AhPIN* genes. These particular *AhPIN* genes displayed a lower motif count compared to other complete PIN genes, suggesting that they might represent fragments associated with a PIN gene or potentially suffer from misannotation (Supplemental Fig. 1). The remaining 16 *AhPIN* genes, which were accurately annotated, were categorized based on their evolutionary relationships and chromosomal locations (Table 1; Fig. 1). To further assess the evolutionary relationships of *AhPIN* genes, an unrooted phylogenetic tree was constructed based on an alignment of 16 AhPIN, eight AtPIN, and 12 OsPIN full-length protein sequences (Fig. 1, Supplemental Table 1). Given the criteria for wheat PIN classifications [22], the AhPIN members were categorized into four subfamilies: I, II, III, and IV. Subfamily I possessed 6 AhPINs and subfamily II contained 2 AhPINs, which were the largest and the smallest group, respectively.

**Table 1:** Characteristic features of PIN gene family in Peanut

No.	PIN names	Peanut gene ID	Start	End	Chromosome	Amino acid length	pI	Mw(kDa)	Subcellular location	GRAVY
1	AhPIN2A	Arahy.Y9MC4Z.1	95,315,993	95,318,730	1	549	9.44	60.4903	Plasma Membrane	0.108
2	AhPIN5A-1	Arahy.KHTN7F.1	39,010,286	39,014,376	3	524	9.22	57.8506	Plasma Membrane	0.237
3	AhPIN3A	Arahy.FCRD5F.1	52,897,656	52,901,853	4	661	6.93	71.5555	Plasma Membrane	0.125
4	AhPIN5A	Arahy.K2GLG1.1	1.19E + 08	1.19E + 08	4	373	8.44	40.8305	Plasma Membrane	0.692
5	AhPIN8A-1	Arahy.13W9YS.1	1.22E + 08	1.22E + 08	4	370	9.3	40.6013	Plasma Membrane	0.504
6	AhPIN1A	Arahy.L76SLB.1	1.02E + 08	1.02E + 08	5	616	8.78	67.3518	Plasma Membrane	0.095
7	AhPIN8A	Arahy.V3Y7Y0.1	1.08E + 08	1.08E + 08	10	368	8.63	40.2731	Plasma Membrane	0.561
8	AhPIN1A-1	Arahy.66N4V9.1	1.12E + 08	1.12E + 08	10	593	8.98	63.7347	Plasma Membrane	0.267
9	AhPIN2B	Arahy.B9QDDU.1	1.49E + 08	1.49E + 08	11	496	8.93	54.7493	Plasma Membrane	0.016
10	AhPIN5B-1	Arahy.HCQJ24.1	41,011,682	41,015,263	13	524	9.22	57.9156	Plasma Membrane	0.233
11	AhPIN3B	Arahy.2QV4RS.1	40,338,987	40,343,406	14	647	6.55	70.0827	Plasma Membrane	0.104
12	AhPIN5B	Arahy.953E9B.1	1.33E + 08	1.33E + 08	14	381	7.56	41.5382	Plasma Membrane	0.691
13	AhPIN8B-1	Arahy.LAPE95.1	1.36E + 08	1.36E + 08	14	328	9.7	36.0163	Plasma Membrane	0.636
14	AhPIN1B	Arahy.72M57I.1	1.42E + 08	1.42E + 08	15	621	8.89	67.6211	Plasma Membrane	0.091
15	AhPIN8B	Arahy.FS1I4N.1	1.33E + 08	1.33E + 08	20	368	8.31	40.3452	Plasma Membrane	0.553
16	AhPIN1B-1	Arahy.E6QCJN.1	1.39E + 08	1.39E + 08	20	593	8.98	63.7617	Plasma Membrane	0.268

As shown in Table 1, the length of AhPIN proteins ranged from 328 (AhPIN8B-1) to 661 (AhPIN3A) amino acids (aa) with an average of 500.75 aa, and corresponding MWs from 36.02 to 71.56 KDa. The pI values varied from 6.55 to 9.70. The GRAVY values of AhPIN proteins ranged from 0.016 to 0.692, all of them were positive, indicating that these proteins shared the characteristic of hydrophobicity. The prediction results of AhPIN subcellar location showed that all of them were found in the plasma membrane.

### Membrane localization of AhPINs

**Fig. 2:**
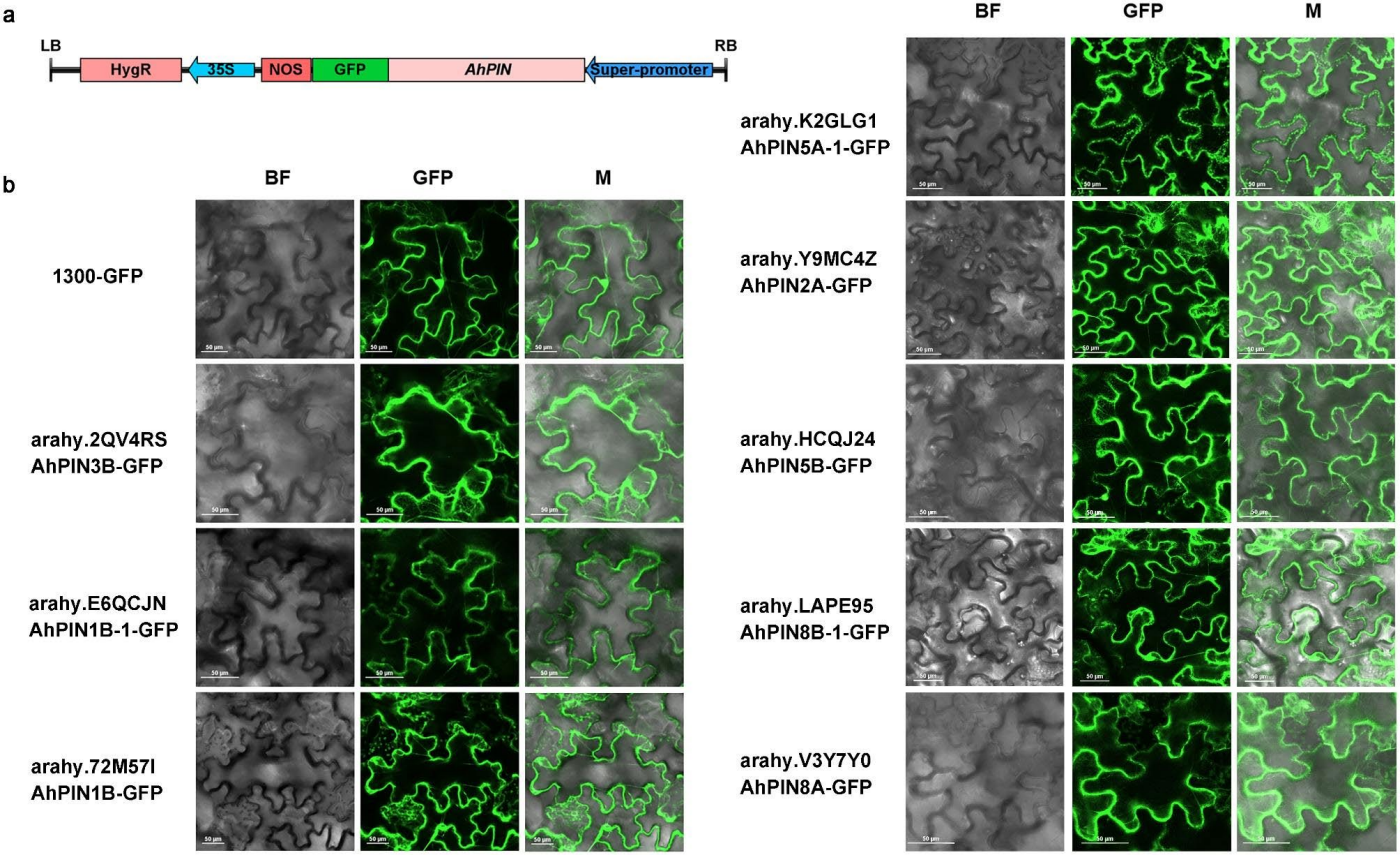
Subcellular localization of eight AhPINs. **(a)** The structure of T-DNA in the vector used in overexpression. **(b)** Subcellular localization of eight AhPINs transiently transferred into tobacco leaves

To detect the subcellular localization of *PIN* family genes in peanut, we constructed a PIN-GFP expression vector using 1300-GFP (Fig. 2a) as the fundamental skeleton. Through an *Agrobacterium*-mediated transient expression system, PIN-GFP was injected into tobacco leaves that were 2–3 weeks old. Compared with 1300-GFP, all PIN-GFPs were expressed in the plasma membrane (Fig. 2b), consistent with the results predicted by the software, indicating that PIN family genes of peanut might have a regulatory role in membrane transport.

### Sequence alignment, gene structure, and motif analysis of AhPINs

**Fig. 3:**
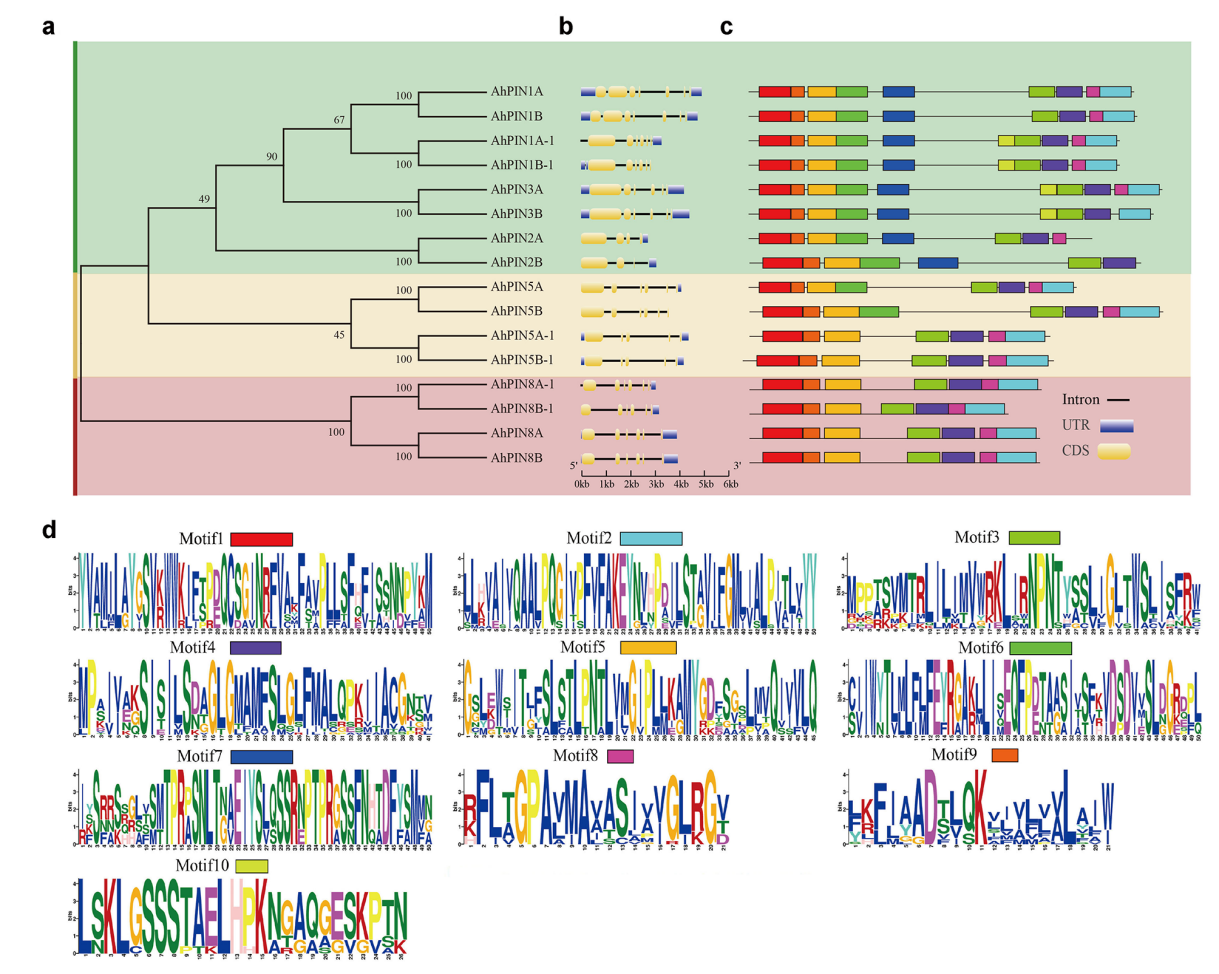
Gene structure and motif analysis of AhPINs. **(a)** Evolutionary tree of AhPINs. **(b)** Gene structure of *AhPINs*. **(c)** Motifs in the protein sequences of AhPINs. **(d)** The sequence logo of motif found in these AhPINs.

The exon-intron structure of subfamilies exhibited significant diversity (Fig. 3a and b). Exon numbers of these *AhPIN*s ranged from 3 (*AhPIN2B*) to 7 (*AhPIN1A* and *AhPIN1B*) and the lengths of exons varied from 35 to 1,662 bp with an average of 712.43 bp. *AhPIN*s clustered in the same group shared similar exon-intron structures, and exon numbers tended to be consistent within a group. Domain analysis showed that all AhPINs had at least one membrane transport domain (PF03547). Among them, AhPIN8A, AhPIN8B and AhPIN8B-1 had two membrane transport domains (Supplemental Table 2).

MEME software was used to explore conserved motifs of AhPIN protein sequences, and ten conserved motifs were predicted and annotated (Fig. 3c and d). The most conserved motifs were 1, 3, 4, 5 and 9, predicted in all *AhPIN* genes (Fig. 3c). Remarkably, certain conserved motifs were identified in specific *AhPIN* genes. For instance, motif 7 was uniquely present in 8 *AhPIN* genes, which shared a closely related evolutionary relationship. Furthermore, motif 1 was found in the N-terminal region, while motif 2 was exclusively distributed in the C-terminal region (Fig. 3c and d).

### Cis-regulatory elements analysis

**Fig. 4:**
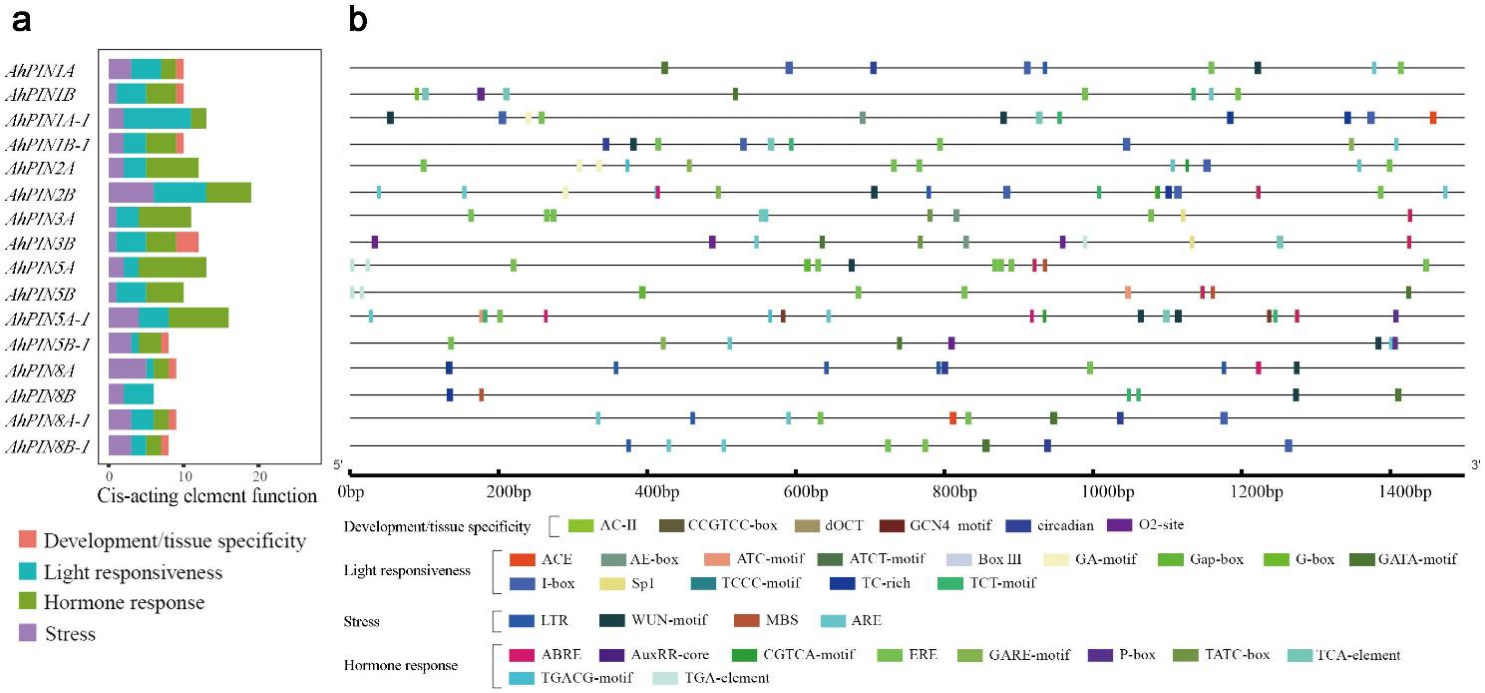
Promoter region (-1500 bp) analysis of *AhPIN*s. **(a)** The number of four types of cis-acting regulatory elements. **(b)** The distribution of cis-acting regulatory elements in the promoter regions. The detailed information of cis-acting regulatory elements could be found in Supplemental Table 3

As the region of the transcription factor binding site that initiates transcription, the promoter is essential in controlling the tissue-specific or stress responses of gene expression. To further infer the regulatory mechanisms and potential functions of *AhPINs*, the upstream 1.5 kb promoter region sequences from the initiation codon were extracted to explore cis-regulatory elements (Fig. 4 and Supplemental Table 3).

Four types of cis-regulatory elements (development/tissue specificity, light responsiveness, stress, and hormone response) were detected (Fig. 4a). The majority of *AhPINs* possessed hormone-, stress- or light-response related cis-elements, except for *AhPIN8B*, which did not possess hormone-response related cis-elements. In total, 8 gibberellin (*GARE-motif, P-box, TATC-box*), 6 MeJA (*CGTCA-motif, TGACG-motif*), 10 ABA (*ABRE*), 5 auxin (*TGA-element*), 31 ethylene (*ERE*), and 7 salicylic acid (*TCA-element*) cis-elements were detected in all *AhPINs* (Fig. 4b and Supplemental Fig. 2). There were four type of abiotic stress cis-elements detected, including *Wun-motif* (*wound-responsive element*, 11 genes), *ARE* (*cis-acting regulatory element essential for anaerobic induction*, 19 genes), *MBS* (*MYB binding site involved in drought-inducibility*, 3 genes) and *LTR* (*low-temperature responsiveness*, 8 genes).

Other cis-elements were also found, including 10 development/tissue specificity-related elements and 58 light-responsive elements (Supplemental Table 3). In total, 50% of the *AhPINs* contained development/tissue specificity-related elements, including *circadian* and *O2-site*. There were 11 types of light-responsive elements observed in the promoter regions of *AhPIN* family genes, with the largest being *I-box*, having 13 in total (Fig. 4 and Supplemental Table 3).

### Chromosomal distribution and gene duplications

The chromosome location information of 16 *AhPIN* genes in peanut was obtained from PeanutBase (https://www.peanutbase.org/home). *AhPIN* genes were distributed on chromosomes 1, 3, 4, 5, 10, 11, 13, 14, 15 and 20 (Supplemental Fig. 3). Chromosomes 4 and 14 contained three genes, while chromosomes 10 and 20 contain two genes and the others contain only one gene.

**Fig. 5:**
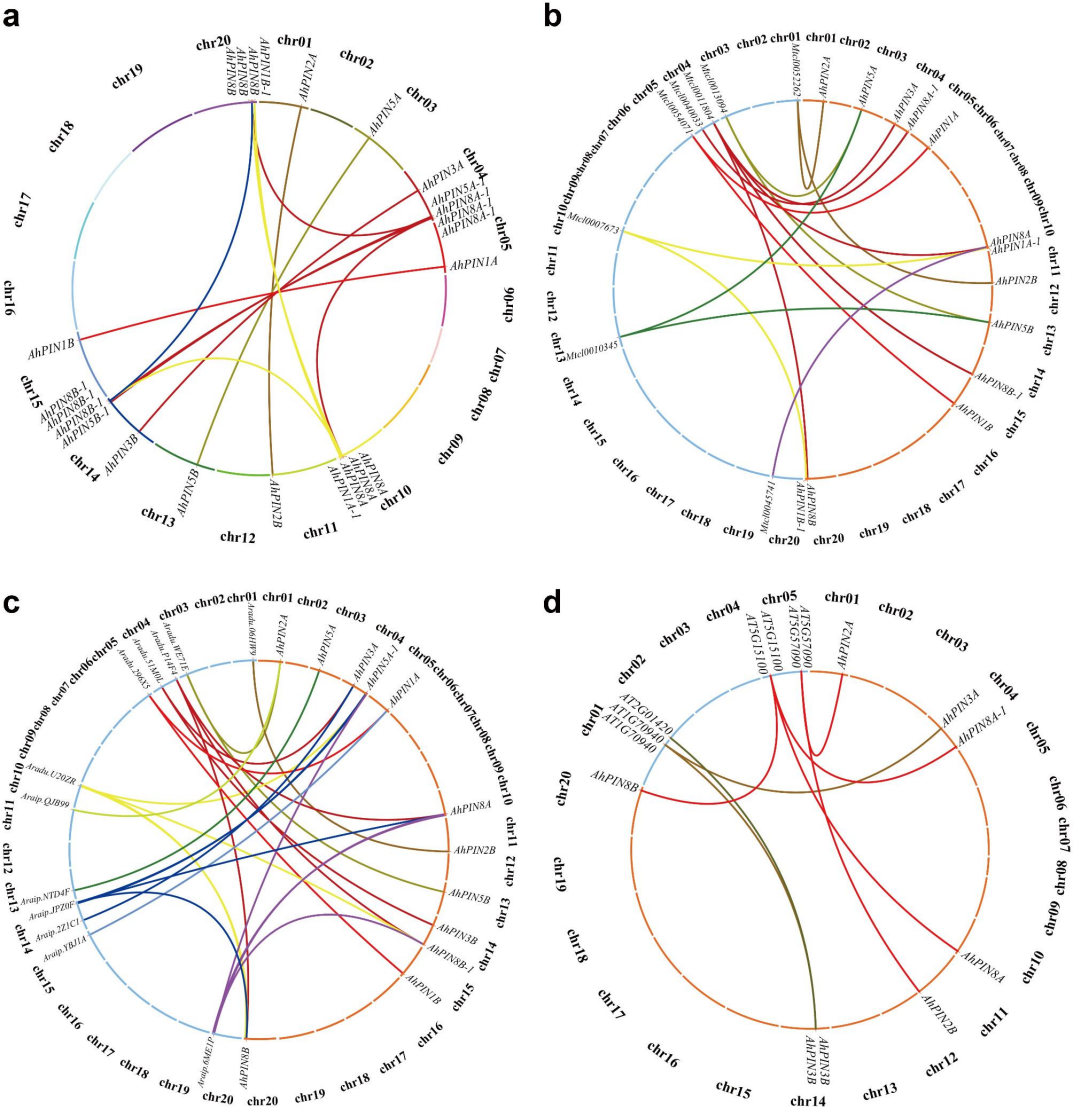
Gene duplications and synteny analysis. **(a)** Gene duplications of *AhPIN*s in *A. hypogaea*. The detailed information of the duplication events could be found in Supplemental Table 4. Synteny analysis of PIN genes between *A. hypogaea* and *A.monticola***(b)**, *A. hypogaea* and *A. duranensis*, as well as *A. ipaënsis***(c)**, and *A. hypogaea* and *A.thaliana***(d)**. The detailed information including the Ka/Ks ratios and estimated divergence time between peanut and other species could be found in Supplemental Table 5

In plant genome evolution, gene duplications (tandem and segmental duplication) lead to gene family expansion. All the identified *AhPIN* gene members were used to ascertain gene duplication in the peanut genome. A total of 12 gene pairs consisting of 16 genes were identified as duplicated, which were associated with segmental duplications (Fig. 5a). Replication events mainly occurred on chromosome 1, 3, 4, 5, 10, 11, 13, 14, 15 and 20. As no tandem duplication was detected, segmental duplication events played a predominant role in the expansion of the PIN gene family. The Ka/Ks ratios for 12 *AhPIN* paralogous gene pairs (Supplemental Table 4) varied from 0.0312 to 0.3983 with an average of 0.1594, suggesting that the PIN gene family has mainly undergone strong purifying selection pressure during the gene evolution process with limited functional divergence occurring after segmental duplication. The divergence time of paralogous genes ranged from 0.8722 to 74.5851 million years ago (Mya) with an average of 26.4339 Mya.

### Synteny analysis for PINs in peanut and its relatives

To explore the evolutionary relationships of *AhPIN* genes, synteny analysis was conducted comparing peanut with other species, specifically *A. monticola*, *A. ipaensis*, *A. duranensis*, and *A. thaliana*. Our analysis revealed the presence of 20, 14, 12 and 8 orthologous pairs between *AhPINs* and other *PIN* genes in *A. monticola, A. ipaensis, A. duranensis*, and *A. thaliana*, respectively (Fig. 5b, c and d).

In order to establish a fundamental framework for understanding the orthologous relationships of PIN genes among peanuts and their relatives, we calculated the Ka/Ks ratios (Supplemental Table 5). Interestingly, in *A. ipaensis* and *A. duranensis*, the progenitors of cultivated peanuts, we observed that 13 out of 14 and all 12 orthologous pairs had Ka/Ks values less than 1, suggesting a history of intense purifying selection during their evolutionary process.

### Expression profiling of peanut *PIN* genes in various tissues

**Fig. 6:**
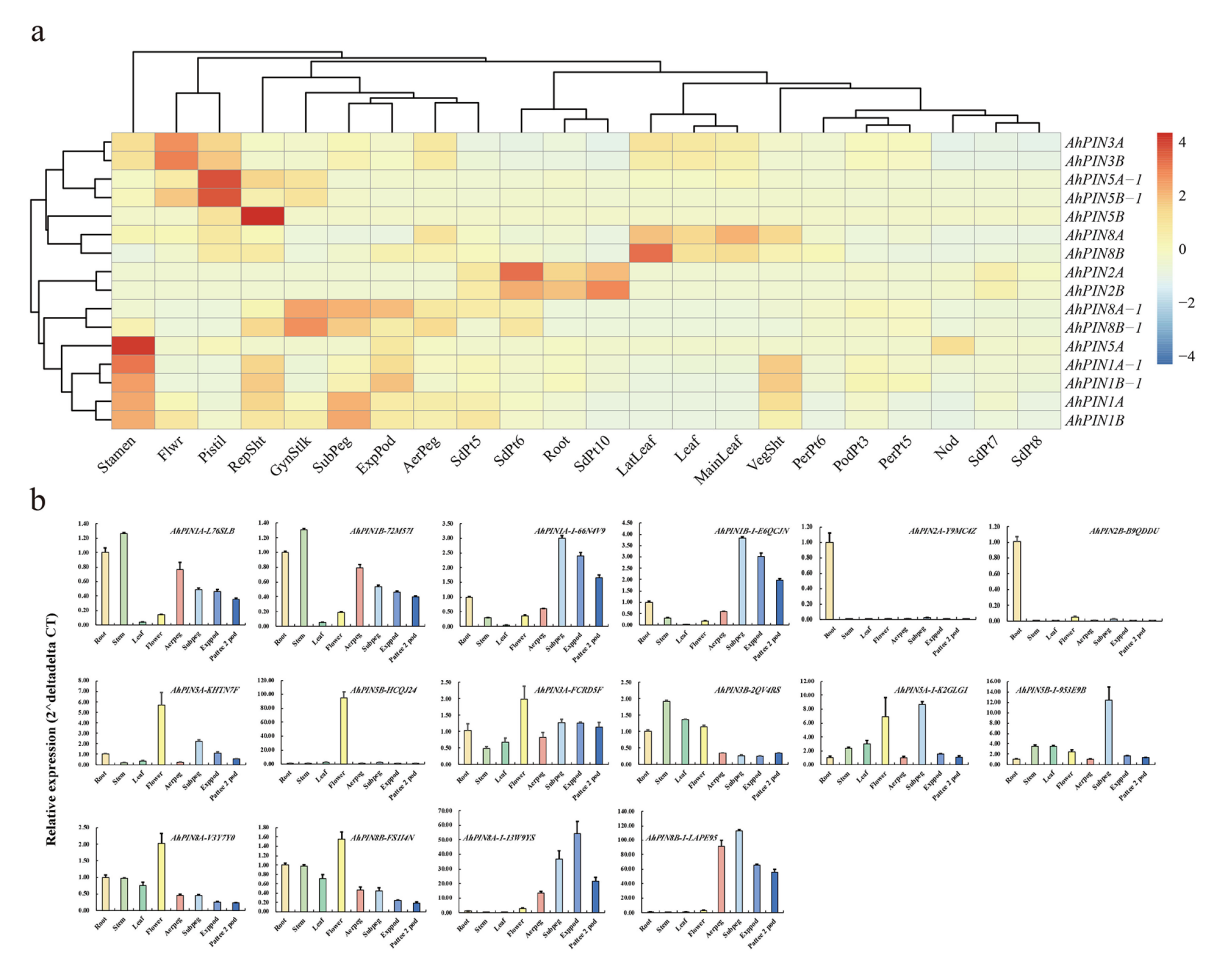
Expression pattern analysis of *AhPIN*s. **(a)** The heatmap presented the expression profiles of *AhPINs* in 22 tissues, utilizing row-wise normalization with Z-scores based on previously published RNA-seq data. The detailed information of the RNA-seq data could be found in Supplemental Fig. 4. The corresponding FPKM value of these *AhPIN*s in 22 tissues could be found in Supplemental Table 6. **(b)** The qRT-PCR showing the expression levels of 16 *AhPIN* genes in root, stem, leaf, flower, and pegs at four distinct developmental stages (Aerpeg: aerial peg; Subpeg: subterranean peg; Exppod: expanded pod; Pattee 2 pod: the expanded pod in the Pattee 2 stage [23]. The corresponding raw data could be found in Supplemental Table 7

Based on the peanut RNA-seq data from publicly available databases, the expression profiles of *AhPINs* in 22 tissues were investigated (accession numbers and sample information of RNA-seq data were listed in Supplemental Fig. 4). Supplemental Table 6 presents the FPKM values for all expressed *AhPIN* genes. Our findings indicate that the majority of the 16 *AhPIN* genes exhibit distinct tissue-specific expression patterns, as revealed by FPKM values normalized using Z-scores (Fig. 6a). Notably, AhPIN3A and AhPIN3B displayed the highest average FPKM levels, with particularly pronounced expression in flowers (Supplemental Table 6). The expression trend of homologous genes was similar. Six *AhPIN* genes, namely *AhPIN1A, AhPIN1B, AhPIN1A-1, AhPIN1B-1, AhPIN3A*, and *AhPIN3B*, were expressed at high levels, with FPKM values exceeding 5 in shoot tissues. *AhPIN1B* exhibited the highest expression in reproductive shoots. *AhPIN2A and AhPIN2B* predominantly expressed in roots. Furthermore, *AhPIN1A, AhPIN1B, AhPIN1A-1* and *AhPIN1B-1* also displayed elevated expression levels in stamen tissue compared to other *AhPIN* genes (Fig. 6a and Supplemental Table 6). *AhPIN2A, AhPIN2B AhPIN5A, AhPIN5B, AhPIN8A, AhPIN8B, AhPIN8A-1 and AhPIN8B-1* displayed comparatively lower average FPKM levels, all falling below 0.5. In every tissue examined, the FPKM levels for these genes did not surpass 1.2 (Supplemental Table 6).

Additionally, qRT-PCR was conducted to assess the relative expression levels of all 16 *AhPIN* genes across eight different tissues: root, stem, leaf, flower, and pegs at four distinct developmental stages (Aerpeg: aerial peg; Subpeg: subterranean peg; Exppod: expanded pod; Pattee 2 pod: the expanded pod in the Pattee 2 stage [23]) (Fig. 6b and Supplemental Table 7). Most of the expression patterns observed were consistent with the RNA-seq data. For instance, *AhPIN2A* and *AhPIN2B* were predominantly expressed in the root across all eight tissues, in line with the RNA-seq data. Similarly, *AhPIN1A, AhPIN1B, AhPIN1A-1*, and *AhPIN1B-1* displayed prominent expression in pegs, aligning with the RNA-seq data, which suggests a potential link to peanut pod development. However, there were also some inconsistencies. For instance, *AhPIN5A* and *AhPIN5B* exhibited higher expression levels in flowers according to the qRT-PCR data, whereas the RNA-seq data indicated their higher expression in stamens and reproductive shoots, respectively (Fig. 6a and b). These disparities may be attributed to variations in the growth environment and the timing of sample collection, which were not entirely consistent with the conditions employed for RNA-seq analysis.

### Protein interaction analysis of *AhPIN* genes

**Fig. 7:**
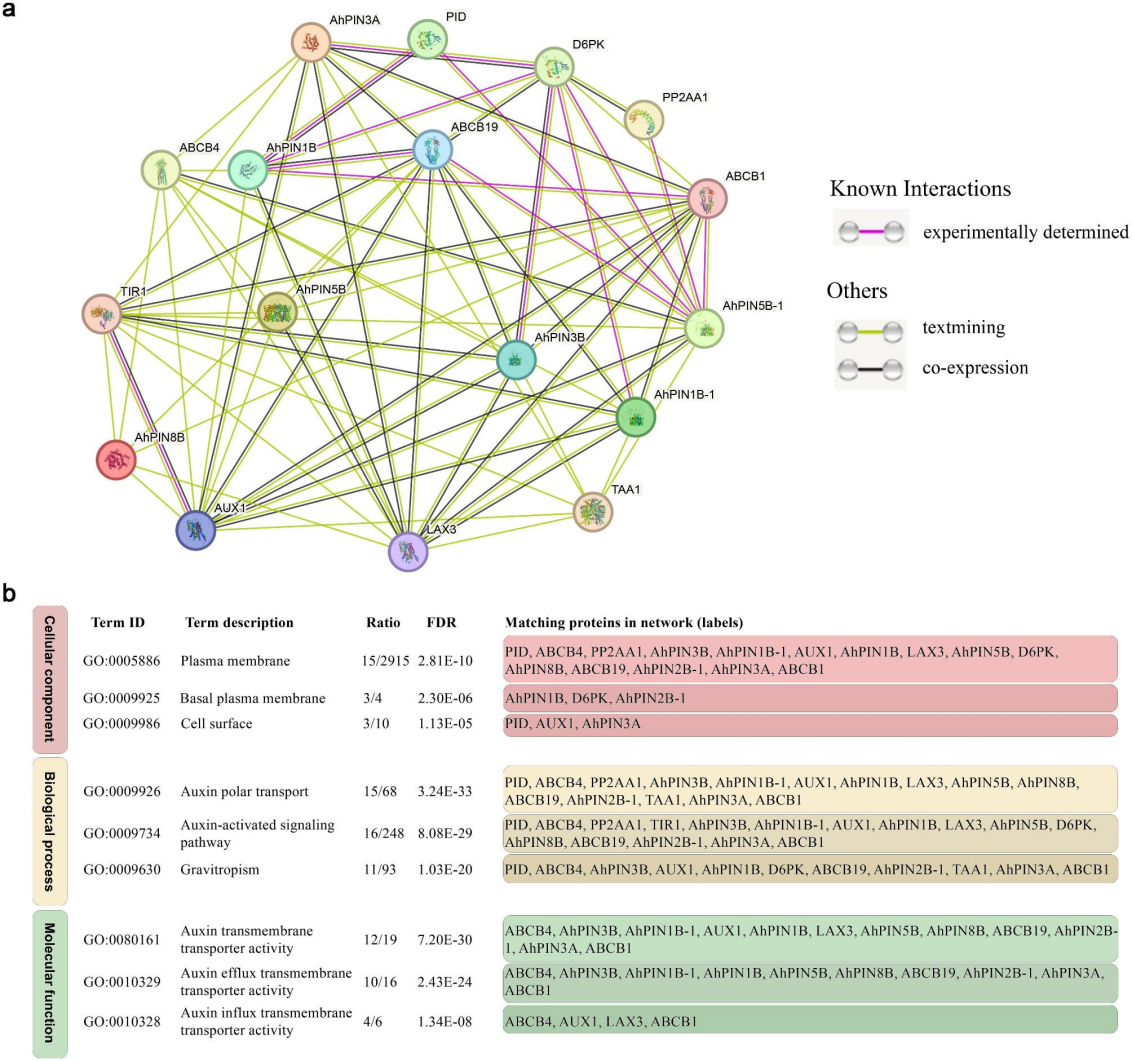
Protein interaction and GO enrichment analysis. **(a)** Protein interactions between AhPINs and other proteins were analyzed using the STRING database, with Arabidopsis as the reference species. The detailed information of these proteins could be found in Supplemental Table 8. **(b)** Gene ontology enrichment of proteins in the interaction network. The detailed information of significant Go terms could be found in Supplemental Table 10

To explore protein-protein interactions involving AhPINs and other proteins, we utilized the STRING database to construct a protein interaction network (Fig. 7a and Supplemental Table 8). This network consisted of 17 nodes and 69 protein interactions, including 7 AhPINs and 10 additional proteins. Remarkably, experimental evidence showed that D6PK (Serine/threonine-protein kinase) interacted with the largest number of AhPINs, namely AhPIN3B, AhPIN3A, AhPIN1B, AhPIN1B-1, and AhPIN5B-1. Additionally, ABCB19 (ATP Binding Cassette Subfamily B Member 19) and AUX1 (Auxin Transporter 1) demonstrated the highest number (4) of co-expressions with AhPINs. Specifically, ABCB19 co-expressed with AhPIN1B, AhPIN3A, AhPIN3B, and AhPIN1B-1, while AUX1 co-expressed with AhPIN1B-1, AhPIN3A, AhPIN3B, and AhPIN5B-1 (Fig. 7a).

Moreover, the gene expression levels of homologous proteins interacting with AhPINs were determined by RNA-seq (Supplemental Table 9). The qRT-PCR was performed for a set of genes, including *ABCB19, AUX1, LAX3 (Auxin transporter-like protein 3), DP6K, PID (Protein kinase PINOID)* and *PP2AA1/RCN1 (Serine/threonine-protein phosphatase 2 A 65 kDa regulatory subunit A alpha isoform)* (Supplemental Fig. 5 and Supplemental Table 7). Although AhABCB19 had a co-expression relationship with AhPIN1B, AhPIN3A, AhPIN3B, and AhPIN1B-1, their expression patterns were not similar. What was consistent, however, was that *AhABCB19, AhPIN3A, AhPIN1B* and *AhPIN1B-1* had higher expression levels in peanut pegs and pods. The proteins with interaction relationships did not have similar expression patterns. Interestingly, AhDP6K and AhRCN1 exhibited highly similar expression patterns across all eight tissues in the qRT-PCR analysis, aligning with the results from the interaction analysis, where the interaction network indicated that they were co-expressed.

### Gene ontology enrichment

GO enrichment analyses were performed to gain insight into the functions of AhPINs and their interacting proteins (Fig. 7b, Supplemental Table 10). The genes were significantly enriched in the plasma membrane (GO:0005886) as a cellular component, auxin polar transport (GO:0009926) as a biological process, and auxin transmembrane transporter activity (GO:0080161) as a molecular function. The cellular component category showed similar results with subcellular localization, indicating that AhPINs were enriched and functioning in the membrane.

## Discussion

### Genetic evolution of PIN genes in cultivated peanut

PINs are plant-specific transmembrane proteins found only in the genomes of land plants [24]. PIN genes in higher plants have relatively high sequence similarity with each other, so they are thought to have evolved from a single ancestor gene. The homolog *PIN* gene can be found in Streptophyte algae, the ancestors of land plants [25]. Because of the endoplasmic reticulum (ER) location in Arabidopsis of AtPIN5, AtPIN8, and PIN proteins from the moss *Physcomitrella patens* [26, 27], ER-located PIN proteins were hypothesized to be ancestral PINs of land plants, while PM (plasma membrane)-located PINs are thought to have been acquired later in evolutionary time [26].

In our previous prediction and experiment on subcellular localization, *PIN* genes in cultivated peanuts were mostly localized in the PM (Table 1; Fig. 1b), so, by assumption, the *PINs* in cultivated peanuts should have been acquired during evolution. Moreover, Ka/Ks values of *PIN* genes between *A. thaliana* and *A. hypogaea* were greater than 1. That is, the substitution times of non-synonymous substitution sites are greater than the substitution times of synonymous substitution sites, which means that selection has changed the protein. Therefore, ​we speculate that PIN proteins evolved in peanuts may have acquired new functions by modifying certain protein structures compared to model plants.

### *PIN* genes have different roles in plants

Auxin is a hormone that regulates nearly all growth and development processes in plants. It is usually synthesized in juvenile tissues such as the tips of roots and stems, and transported to target tissues via vascular bundle tissue or polar transport [28].​ A variety of auxin transporters are involved in auxin polar transport, such as AUXIN1/LIKE-AUX1 (AUX/LAX), PIN, ATP-binding cassette (ABC) transporter, nitric acid transporter 1.1 (NRT1.1), PIN-like transporter (PIL), and WALLS ARE THIN 1 (WAT1) [29]. In our interaction network, proteins experimentally interacting with AhPINs included D6PK, ABCB19, PID, PP2AA1 and ABCB1 (Fig. 7a). D6PK is a membrane-bound protein that is associated with either the basal domain of the plasma membrane or endomembrane, and can directly phosphorylate PINs. D6PK-dependent PIN phosphorylation is a prerequisite for PIN-mediated auxin transport [30]. The Arabidopsis ATP-binding cassette B19 (ABCB19, P-glycoprotein19) transporter functions coordinately with ABCB1 and PIN1 to mediate long-distance transport of the phytohormone auxin from the shoot to root apex [31]. PP2A and PINOID both partially colocalize with PINs and act antagonistically on the phosphorylation state of their central hydrophilic loop, thereby mediating PIN apical-basal polar targeting, which mediates differential growth, tissue patterning, and organogenesis [32].

In Arabidopsis, the function of the *PIN* gene has been well studied and eight *PIN* genes have different functions during auxin signaling mediated development and growth. PIN1 is involved in vascular development [33]; PIN2 is mainly expressed in the elongation region of root tips and is involved in root geotropism [12]; PIN3 and PIN4 have similar functions and act in phototaxis and root development [34–37]; PIN5 mediates subcellular compartmentalization of auxin localized in the ER [26]; PIN6 has dual localization in the PM and ER to regulate auxin transport in the PM and intracellular auxin homeostasis [37]; PIN7 negatively controls radial root growth [38]; and PIN8 is an ER-localized protein, which is mainly involved in reproductive development [27, 39].

In addition to Arabidopsis, some progress has been made in determining the role of PIN in other plants. In maize, ZmPIN1 protein is required for embryogenesis [40]. In cotton, PIN protein functions in regulating fiber growth [41], and in rice, OsPIN2 is expressed in root epidermis and cortex cells to regulate geotropic response and root structure [42]. In soybean, GmPIN is involved in nodule development [43]. In peanut, some *PIN* genes associated with peanut peg geotropism and pod development were also identified from our unpublished studies. ​Compared to Arabidopsis, cultivated peanuts have more than three times the number of PINs (Table 1), and ​their function is most likely enriched by evolution.​ Also, peanut has the special feature of underground pods. Therefore, additional studies of PIN function in peanuts will likely provide new insights to reveal more fascinating and novel functions of PINs.

### *PIN* genes involved in nitrogen fixation by nodules

Legumes can interact with rhizobia to form nodules. Using nodules, legumes can convert nitrogen from the air into ammonium, which can be taken up by plants. This mode of nitrogen uptake is known as biological nitrogen fixation [44]. On an average, 50–60% of soybean N demand is met by biological N2 fixation [45]. In peanut, root nodules are the greatest N source, accounting for about 50.0%, and the N supply proportion from root nodule, soil, and N fertilizer was 5:3:2 [46]. ​The formation and development of root nodules involves complex regulatory mechanisms in which auxin, as well as PINs, are suspected to have an essential role [47, 48]. ​In soybeans, GmPIN1 is specifically expressed in the root nodule primordium region, and root nodule primordium development is inhibited in GmPIN1 overexpressed plants. GmPIN9d protein accumulates in the vascular bundle between roots and nodules, and synergizes with GmPIN1 to regulate auxin polar transport to promote the growth of nodules ​ [40]. ​In *Medicago truncatula*, the number of root nodules in MtPIN2, MtPIN3, and MtPIN4-silenced plants (via RNA interference) decreased significantly [49]. ​In our study, there were ten AhPIN genes expressed in 25-day nodules (Fig. 6 and Supplemental Table 6). Their expression levels were not high, possibly because the root nodules had formed and thus auxin transport activity was slowed. It is possible that the detection of newly developed nodules will result in higher expression.​ However, the relationship between PIN genes and nodule formation is unclear, and there are numerous gaps to be filled. In the last few years, gene editing technology has profoundly changed the course of breeding, and perhaps in the near future, we may be able to improve the nitrogen fixing ability of legumes by altering, for example, the expression of PIN genes.

## Conclusion

​In this study, 16 *PIN* genes in cultivated peanuts were identified and their subcellular locations, gene structure, cis-elements, evolutionary relationships, transcriptional expression, and interaction networks were analyzed to give a comprehensive understanding of *PIN*s in peanuts. ​This work lays a foundation for investigating auxin transport and *PIN* related gene function in peanuts.​.

### Methods

### Identification and prediction of *AhPIN* genes in *Arachis hypogaea*

Protein sequences of *A. hypogaea* cv. Tifrunner were acquired from PeanutBase (https://www.peanutbase.org/home). Protein sequences of Arabidopsis PIN protein sequences downloaded from the Arabidopsis information resource (TAIR, http://www.arabidopsis.org) were used as query sequences for BLASTP searches against *A. hypogaea* proteins to obtain the *AhPIN* genes. The reliability of these potential proteins was confirmed by CDD (Conserved Domain Database: http://www.ncbi.nlm.nih.gov/Structure/cdd/wrpsb.cgi) analysis. The isoelectric point (pI), molecular weight (MW), subcellular location, and GRAVY (grand average of hydropathy) of AhPIN proteins were predicted by the ProtParam online tool (https://web.expasy.org/protparam/).

### Phylogenetic, protein structure, and promoter analysis of the AhPINs

Protein sequences of AtPIN and OsPIN were downloaded from TAIR and https://www.ricedata.cn/gene/. A phylogenetic tree was constructed using the maximum-likelihood (ML) method in MEGA V6.0. The gene structure of *AhPINs* was revealed by comparing coding domain sequences (CDS) and DNA sequences through the GSDS online tool (http://gsds.cbi.pku.edu.cn). Domain organization of *AhPIN* genes was predicted using HMMER (http://www.hmmer.org) with default parameters. Conserved motifs were determined using MEME (http://meme.nbcr.net/meme/intro.html). PlantCare (http://bioinformatics.psb.ugent.be/webtools/plantcare/html/) was used to identify putative cis- regulatory elements.

### Chromosomal mapping and gene duplication analysis

Mapchart (https://www.wur.nl/en/show/Mapchart.htm) was used to map the genes to chromosomes. Genomic data of *Arachis duranensis* and *Arachis ipaensis* was downloaded from PeanutBase. Interspecific and intraspecific syntenic analyses were performed using Multiple Collinearity Scan toolkit (MCScanX) with default parameters. The nonsynonymous substitution rate (Ka) to synonymous substitution rate (Ks) ratios was calculated by Kakas Calculator 2.0 [50]. The divergence times in millions of years ago (Mya) were estimated by the formula: T = Ks/ (2 × 6.1 × 10^− 9^) ×10^− 6^ [51].

### Plant material and gene expression analysis

Peanut pegs of ‘Tifrunner’ were collected at four periods (Aerpeg: aerial peg; Subpeg: subterranean peg; Exppod: expanded pod; Pattee 2 pod: the expanded pod in stage of Pattee 2 [23]). Roots, stems, leaves and flowers were taken from peanut seedlings planted in the experimental station of Peking University Institute of Advanced Agricultural Science, Weifang, China. Total RNA was isolated using RNAprep Pure (TIANGEN, DP441). EasyScript® One-Step gDNA Removal and cDNA Synthesis SuperMix (TransGen, AE311-02) were used for cDNA synthesis. For qRT-PCR, SuperReal Premix PLUS-SYBR GREEN (TIANGEN, FP205) was used to perform the reaction on an Applied Biosystems QuantStudio 5, with the following conditions: initial denaturation at 95℃ for 30 s, 40 cycles of 95℃ for 10 s, 56℃ for 20 s, and 72℃ for 20 s, then a melting curve cycle from 65–95℃. *AhTubulin* was used as an internal control gene and qRT-PCR was conducted with three biological replicates for each sample. Relative gene expression was determined using the 2−△△CT method [52], with root samples serving as the calibrator [53, 54]. All primers are presented in Supplemental Table 11.

The RNA-seq data of 22 tissues from a previous study [55] were used to investigate the expression profiles of *AhPIN* genes. The accession numbers of raw data and sample information of RNA-seq data are listed in Supplemental Fig. 4. The raw sequencing reads were downloaded from the NCBI (https://www.ncbi.nlm.nih.gov/), the adapter sequences and low-quality reads (Q < 20) were removed using Trimmomatic software [56]. All the clean reads were aligned to Tifrunner peanut genome (https://www.peanutbase.org) using HISAT2 (version 2.1.0) [57] with default parameter. Read counts number was calculated using HTSeq (Version 0.7.1) [58] and gene expression levels were estimated using RPKM values (Reads Per Kilobase transcriptome per millionreads).

### *AhPIN* gene cloning and subcellular localization

We selected five *AhPIN* genes from PeanutBase and cloned the coding regions. The *AhPINs* were inserted into an overexpression vector (pCAMBIA1300-GFP). The fusion vector (pCAMBIA1300-AhPINS-GFP) and the negative control (pCAMBIA1300-GFP) were transformed into *Agrobacterium tumefaciens* strain GV3101 and then injected into tobacco leaves via an *Agrobacterium*-mediated transformation system [59]. The GFP fluorescence signal was acquired using a Nikon ECLIPSE Ti2 confocal microscope. We used NIS-Elements Viewer 5.21 to process the images. Primer sequences of the 1300-GFP are shown in Supplemental Table 12.

### Protein interaction prediction and gene ontology enrichment analysis

The STRING database (https://cn.string-db.org) was used to construct the protein interaction network, with Arabidopsis as the reference species. A minimum required interaction score of high confidence (0.700) and a p-value threshold of < 1e-10 were applied. Additionally, GO enrichment analyses were conducted using AgriGO with default parameters [60].

### Electronic supplementary material

Below is the link to the electronic supplementary material.

Supplementary Material 1: Figure 1. Comparison of motifs among AtPINs, OsPINs, and AhPINs. Figure 2. The number of cis-acting regulatory elements in the promoter region of *AhPINs* gene. Figure 3. Chromosomal distribution of *AhPINs*. Figure 4. Accession number and samples information of RNA-seq data used in this study. Figure 5. The qRT-PCR showing the expression levels of six proteins that interacted with AhPINs within the interaction network. Table 1. PIN proteins from Arabidopsis, rice and peanut. Table 2. Domain of AhPIN genes predicted using HMMER with default parameters. Table 3. Characteristics of cis-acting regulatory elements in the promoter regions of the AhPIN genes. Table 4. The duplication information of AhPINs. Table 5. The Ka/Ks ratios and estimated divergence time between peanut and other species. Table 6. FPKM value of AhPINs. Table 7. Raw data of qRT-PCR. Table 8. Protein interaction analysis of AhPINs proteins. Table 9. The homologous peanut proteins within the network and FPKM values in the RNA-seq data. Table 10. Significant Go term of proteins in the interaction network. Table 11. Primers for qRT-PCR. Table 12. Primers for constructing the 1300-GFP vector

Supplementary Material 2: Table 1. PIN proteins from Arabidopsis, rice and peanut used to generate phylogenetic tree

## Data Availability

The RNA-Seq data used in this study derived from NCBI (http://www.ncbi.nlm.nih.gov/) under BioProject PRJNA291488. Accession number: SRR2135539-SRR2135548, SRR2135552, SRR2135554-SRR2135557, SRR2135560, SRR2135586, SRR2135589, SRR2135593, SRR2135597, SRR2135601, SRR2140727-SRR2140728, SRR2140738-SRR2140745, SRR2140748-SRR2140751, SRR2140804-SRR2140805, SRR2141559, SRR2141618-SRR2141620, SRR2141679, SRR2141695-SRR2141705.

## References

[1] Enders TA, Strader LC (2015). Auxin activity: past, present, and future. Am J Bot.

[2] Hammes UZ, Murphy AS, Schwechheimer C (2022). Auxin transporters—a biochemical view. Cold Spring Harb Perspect Biol.

[3] Kramer EM, Bennett MJ (2006). Auxin transport: a field in flux. Trends Plant Sci.

[4] Petrásek J, Friml J. Development. Auxin transport routes in plant development. 2009. 10.1242/dev.030353 19633168

[5] Mohanta TK, Bashir T, Hashem A, Abd-Allah EF, Khan AL, Al-Harrasi AS (2018). Molecular players of auxin transport systems: advances in genomic and molecular events. J Plant Interact.

[6] Muday GK, DeLong A (2001). Polar auxin transport: controlling where and how much. Trends Plant Sci.

[7] Marhava P (2022). Recent developments in the understanding of PIN polarity. New Phytol.

[8] Geisler M, Wang B, Zhu J (2014). Auxin transport during root gravitropism: transporters and techniques. Plant Biol (Stuttg).

[9] Han H, Adamowski M, Qi L, Alotaibi SS, Friml J (2021). PIN-mediated polar auxin transport regulations in plant tropic responses. New Phytol.

[10] Paponov IA, Teale WD, Trebar M, Blilou I, Palme K (2005). The PIN auxin efflux facilitators: evolutionary and functional perspectives. Trends Plant Sci.

[11] Tan C, Wang H, Zhang Y, Qi B, Xu G, Zheng H (2011). A proteomic approach to analyzing responses of *Arabidopsis thaliana* root cells to different gravitational conditions using an agravitropic mutant, pin2 and its wild type. Proteome Sci.

[12] Müller A, Guan C, Gälweiler L, Tänzler P, Huijser P, Marchant A (1998). AtPIN2 defines a locus of Arabidopsis for root gravitropism control. EMBO J.

[13] Baster P, Robert S, Kleine-Vehn J, Vanneste S, Kania U, Grunewald W (2013). SCFTIR1/AFB‐auxin signalling regulates PIN vacuolar trafficking and auxin fluxes during root gravitropism. EMBO J.

[14] Kleine-Vehn J, Ding Z, Jones AR, Tasaka M, Morita MT, Friml J (2010). Gravity-induced PIN transcytosis for polarization of auxin fluxes in gravity-sensing root cells. Proc Natl Acad Sci U S A.

[15] Kumar R, Pandey MK, Roychoudhry S, Nayyar H, Kepinski S, Varshney RK (2019). Peg biology: deciphering the molecular regulations involved during peanut peg development. Front Plant Sci.

[16] Su SH, Gibbs NM, Jancewicz AL, Masson PH (2017). Molecular mechanisms of root gravitropism. Curr Biol.

[17] Moctezuma E (2003). The peanut gynophore: a developmental and physiological perspective. Can J Bot.

[18] Moctezuma E, Feldman LJ (1999). Auxin redistributes upwards in graviresponding gynophores of the peanut plant. Planta.

[19] Li H, Chen X, Zhu F, Liu H, Hong Y, Liang X (2013). Transcriptome profiling of peanut (*Arachis hypogaea*) gynophores in gravitropic response. Funct Plant Biol.

[20] Zhao C, Zhao S, Hou L, Xia H, Wang J, Li C (2015). Proteomics analysis reveals differentially activated pathways that operate in peanut gynophores at different developmental stages. BMC Plant Biol.

[21] Chen X, Yang Q, Li H, Li H, Hong Y, Pan L (2016). Transcriptome-wide sequencing provides insights into geocarpy in peanut (*Arachis hypogaea* L). Plant Biotechnol J.

[22] Kumar M, Kherawat BS, Dey P, Saha D, Singh A, Bhatia SK (2021). Genome-wide identification and characterization of PIN-FORMED (PIN) gene family reveals role in developmental and various stress conditions in *Triticum aestivum* L. Int J Mol Sci.

[23] Pattee H, Johnss EB, Singleton JA, Sanders TH (1974). Composition changes of peanut fruit parts during maturation. Peanut Sci.

[24] Křeček P, Skůpa P, Libus J, Naramoto S, Tejos R, Friml J (2009). The PIN-FORMED (PIN) protein family of auxin transporters. Genome Biol.

[25] Zazimalova E (2007). Polar transport of the plant hormone auxin – the role of PIN-formed (PIN) proteins. Cell Mol Life Sci.

[26] Mravec J, Skůpa P, Bailly A, Hoyerová K, Křeček P, Bielach A (2009). Subcellular homeostasis of phytohormone auxin is mediated by the ER-localized PIN5 transporter. Nature.

[27] Dal Bosco C, Dovzhenko A, Liu X, Woerner N, Rensch T, Eismann M (2012). The endoplasmic reticulum localized PIN8 is a pollen-specific auxin carrier involved in intracellular auxin homeostasis. Plant J.

[28] Swarup R, Péret B (2012). AUX/LAX family of auxin influx carriers—An overview. Front. Plant Sci.

[29] Zhou JJ, Luo J (2018). The PIN-FORMED auxin efflux carriers in plants. Int J Mol Sci.

[30] Barbosa IC, Zourelidou M, Willige BC, Weller B, Schwechheimer C (2014). D6 PROTEIN KINASE activates auxin transport-dependent growth and PIN-FORMED phosphorylation at the plasma membrane. Dev Cell.

[31] Yang H, Richter GL, Wang X, Młodzińska E, Carraro N, Ma G (2013). Sterols and sphingolipids differentially function in trafficking of the Arabidopsis ABCB19 auxin transporter. Plant J.

[32] Michniewicz M, Zago MK, Abas L, Weijers D, Schweighofer A, Meskiene I (2007). Antagonistic regulation of PIN phosphorylation by PP2A and PINOID directs auxin flux. Cell.

[33] Galweiler L, Guan C, Muller A, Wisman E, Mendgen K, Yephremov A (1998). Regulation of polar auxin transport by AtPIN1 in Arabidopsis vascular tissue. Science.

[34] Friml J, Benková E, Blilou I, Wisniewska J, Hamann T, Ljung K (2002). AtPIN4 mediates sink-driven auxin gradients and root patterning in Arabidopsis. Cell.

[35] Keuskamp DH, Pollmann S, Voesenek LA, Peeters AJ, Pierik R (2010). Auxin transport through PIN-FORMED 3 (PIN3) controls shade avoidance and fitness during competition. Proc Natl Acad Sci U S A.

[36] Zadnikova P, Petrasek J, Marhavy P, Raz V, Vandenbussche F, Ding Z (2010). Role of PIN-mediated auxin efflux in apical hook development of Arabidopsis thaliana. Development.

[37] Simon S, Skůpa P, Viaene T, Zwiewka M, Tejos R, Klíma P (2016). PIN6 auxin transporter at endoplasmic reticulum and plasma membrane mediates auxin homeostasis and organogenesis in Arabidopsis. New Phytol.

[38] Rosquete MR, Waidmann S, Kleine-Vehn J (2018). PIN7 auxin carrier has a preferential role in terminating radial root expansion in Arabidopsis thaliana. Int J Mol Sci.

[39] Ding Z, Wang B, Moreno I, DupláKová N, Simon S, Carraro N (2012). ER-localized auxin transporter PIN8 regulates auxin homeostasis and male gametophyte development in Arabidopsis. Nat Commun.

[40] Forestan C, Varotto S (2012). The role of PIN auxin efflux carriers in polar auxin transport and accumulation and their effect on shaping maize development. Mol Plant.

[41] Zhang Y, He P, Yang Z, Huang G, Wang L, Pang C (2017). A genome-scale analysis of the PIN gene family reveals its functions in cotton fiber development. Front Plant Sci.

[42] Wang L, Guo M, Li Y, Ruan W, Mo X, Wu Z (2018). LARGE ROOT ANGLE1, encoding OsPIN2, is involved in root system architecture in rice. J Exp Bot.

[43] Gao Z, Chen Z, Cui Y, Ke M, Xu H, Xu Q (2021). GmPIN-dependent polar auxin transport is involved in soybean nodule development. Plant Cell.

[44] Suzaki T, Yoro E, Kawaguchi M (2015). Leguminous plants: inventors of root nodules to accommodate symbiotic bacteria. Int Rev Cell Mol Biol.

[45] Salvagiotti F, Cassman KG, Specht JE, Walters DT, Weiss A, Dobermann A (2008). Nitrogen uptake, fixation and response to fertilizer n in soybeans: a review. Field Crops Res.

[46] Wu Z, Zheng Y, Chen D, Wang C, Sun X, Li X (2016). Supply characteristics of different nitrogen sources and nitrogen use efficiency in peanut. Chin J Oil Crop Sci.

[47] Pacios-Bras C, Schlaman HR, Boot K, Admiraal P, Mateos Langerak J, Stougaard J (2003). Auxin distribution in Lotus japonicus during root nodule development. Plant Mol Biol.

[48] Sańko-Sawczenko I, Dmitruk D, Łotocka B, Różańska E, Czarnocka W (2019). Expression analysis of PIN genes in root tips and nodules of Lotus japonicus. Int J Mol Sci.

[49] Huo X, Schnabel E, Hughes K, Frugoli J (2006). RNAi phenotypes and the localization of a protein: GUS fusion imply a role for Medicago truncatula PIN genes in nodulation. J Plant Growth Regul.

[50] Wang D, Zhang Y, Zhang Z, Zhu J, Yu J (2010). KaKs_Calculator 2.0: a toolkit incorporating gamma-series methods and sliding window strategies. Genomics Proteom Bioinf.

[51] Lynch M, Conery JS (2000). The evolutionary fate and consequences of duplicate genes. Science.

[52] Livak KJ, Schmittgen TD (2001). Analysis of relative gene expression data using real-time quantitative PCR and the 2^–△△CT^ method. Methods.

[53] Chen M, Li K, Li H, Song CP, Miao Y (2017). The glutathione peroxidase Gene Family in *Gossypium hirsutum*: genome-wide identification, classification, Gene expression and functional analysis. Sci Rep.

[54] Ma J, Dai JX, Liu XW, Lin D (2021). Genome-wide and expression analysis of B-box gene family in pepper. BMC Genomics.

[55] Clevenger J, Chu Y, Scheffler B, Ozias-Akins P (2016). A developmental transcriptome map for allotetraploid Arachis hypogaea. Front Plant Sci.

[56] Bolger AM, Lohse M, Usadel B (2014). Trimmomatic: a flexible trimmer for Illumina sequence data. Bioinformatics.

[57] Kim D, Paggi JM, Park C, Bennett C, Salzberg SL (2019). Graph-based genome alignment and genotyping with HISAT2 and HISAT-genotype. Nat Biotechnol.

[58] Anders S, Pyl PT, Huber W (2015). HTSeq–a Python framework to work with high-throughput sequencing data. Bioinf (Oxford England).

[59] Buschmann H (2016). Plant Cell Division analyzed by Transient Agrobacterium-Mediated Transformation of Tobacco BY-2 Cells. Methods Mol Biol.

[60] Tian T, Liu Y, Yan H, You Q, Yi X, Du Z (2017). agriGO v2. 0: a GO analysis toolkit for the agricultural community, 2017 update. Nucleic Acids Res.

