# Supplementary material for: Genome-wide analysis of PIN genes in cultivated peanuts (Arachis hypogaea L.): identification, subcellular localization, evolution, and expression patterns
Source: BMC Genomics. 2023 Oct 21;24:629. doi: 10.1186/s12864-023-09723-5 (PMC10590530; doi:10.1186/s12864-023-09723-5)
Supplement: Supplementary file 2 — Supplementary Material 2: Table 1. PIN proteins from Arabidopsis, rice and peanut used to generate phylogenetic tree [file 12864_2023_9723_MOESM2_ESM.docx]

**Supplemental information:**

**Genome-Wide Analysis of *PIN* Genes in Cultivated Peanuts (*Arachis hypogaea* L.): Identification, Subcellular location, Evolution, and Expression patterns**

Jianxin Bian^1, †^, Yuanyuan Cui^1, †^, Jihua Li^1^, Yu Guan^1^, Shuhua Tian^1^, Xiaoqin Liu^1*^

^1^ Peking University Institute of Advanced Agricultural Science, Shandong Laboratory for Advanced Agricultural Sciences at Weifang, Shandong 261325, China

^†^These authors contributed equally to this work

* Correspondence: xiaoqin.liu@pku-iaas.edu.cn

**Supplemental Figure 1 Comparison of motifs among AtPINs, OsPINs, and AhPINs**

**
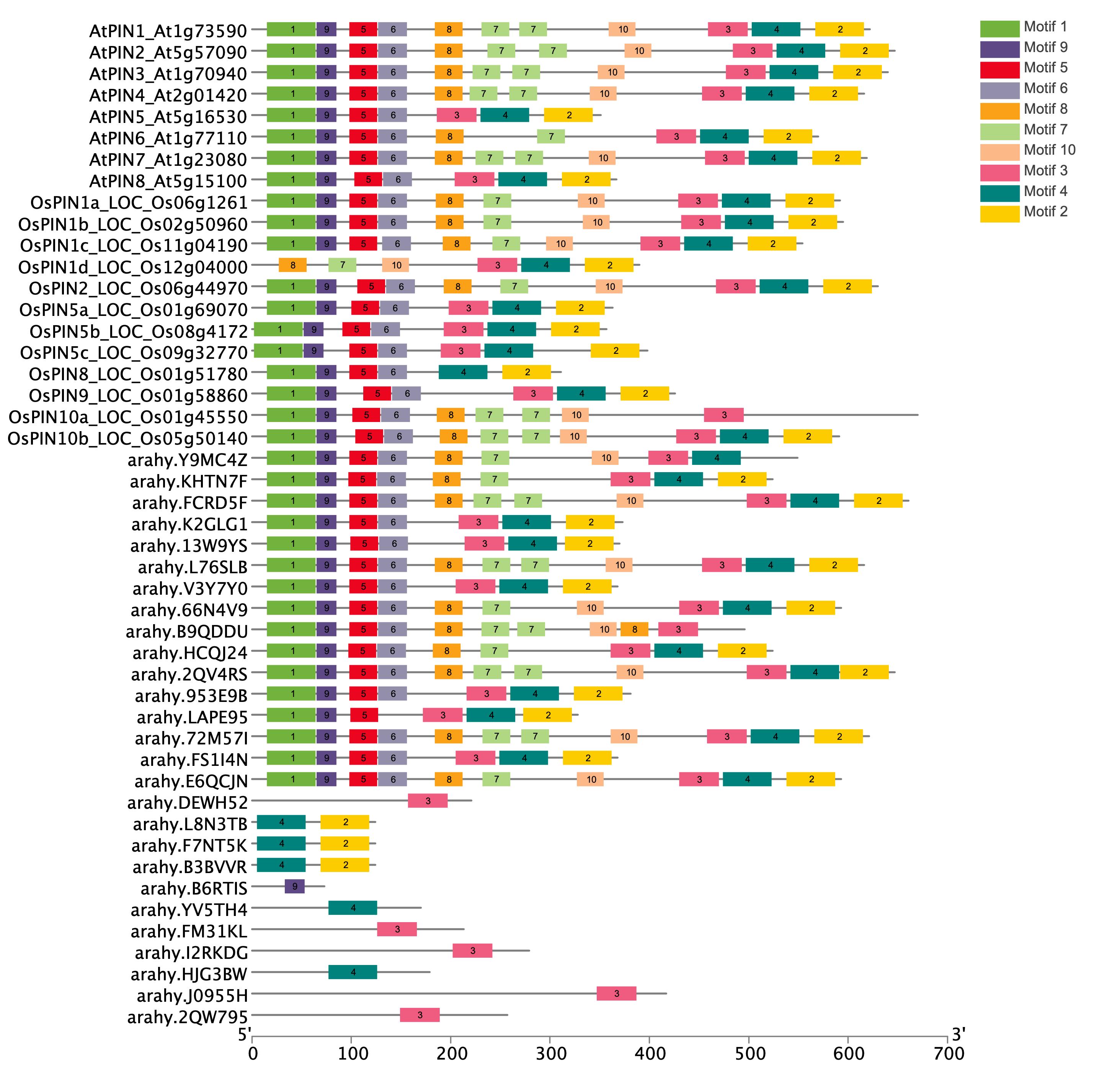
**

**Supplemental Figure 2 The number of cis-acting regulatory elements in the promoter region of *AhPIN* gene.**

**
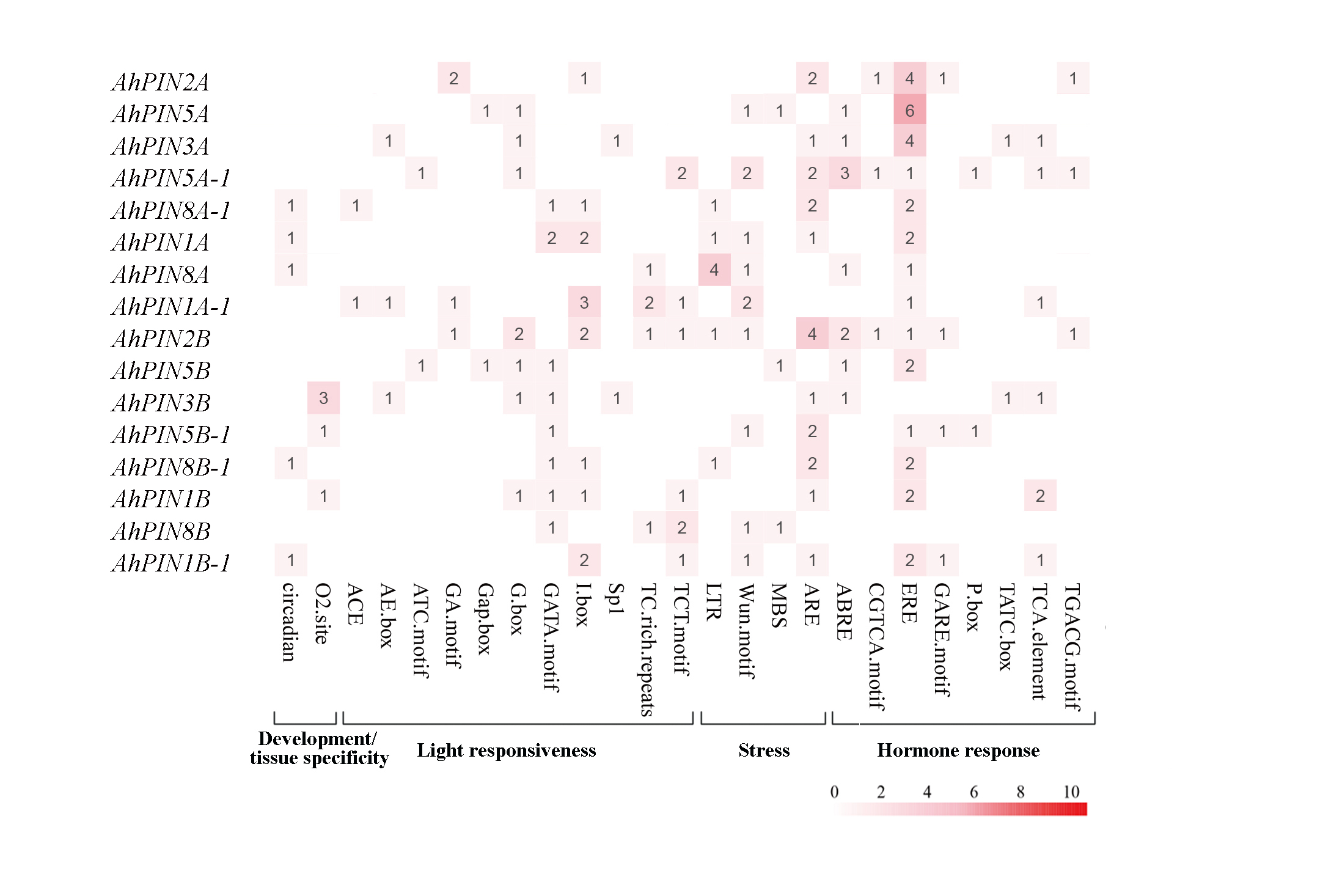
**

**Supplemental Figure 3 Chromosomal distribution of *AhPIN*s.**

**
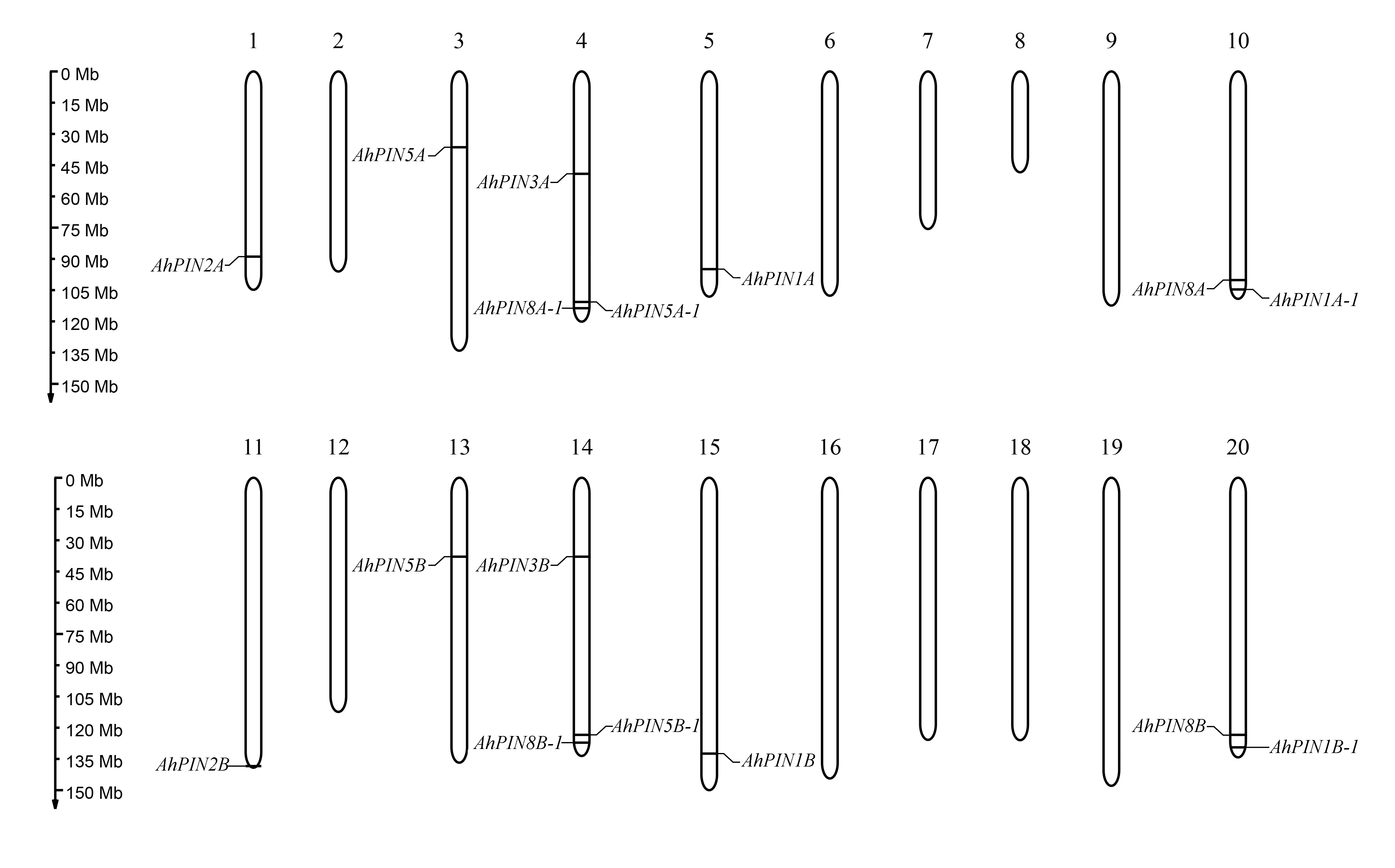
**

**Supplemental Figure 4 Accession number and samples information of RNA-seq data used in this study.**

**
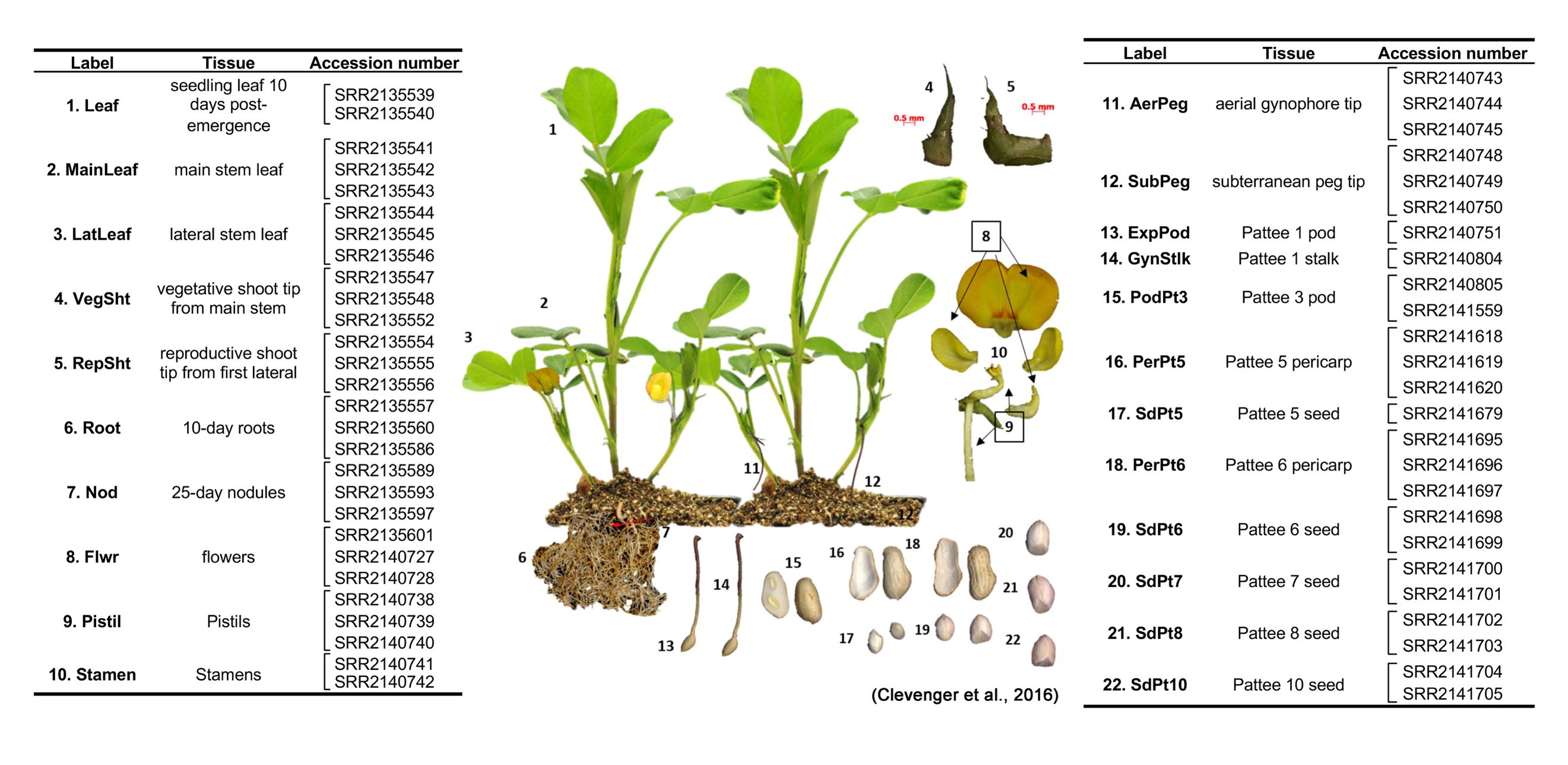
**

**Supplemental Figure 5 The qRT-PCR showing the expression levels of** **six proteins that interacted with AhPINs within the interaction network.**

**
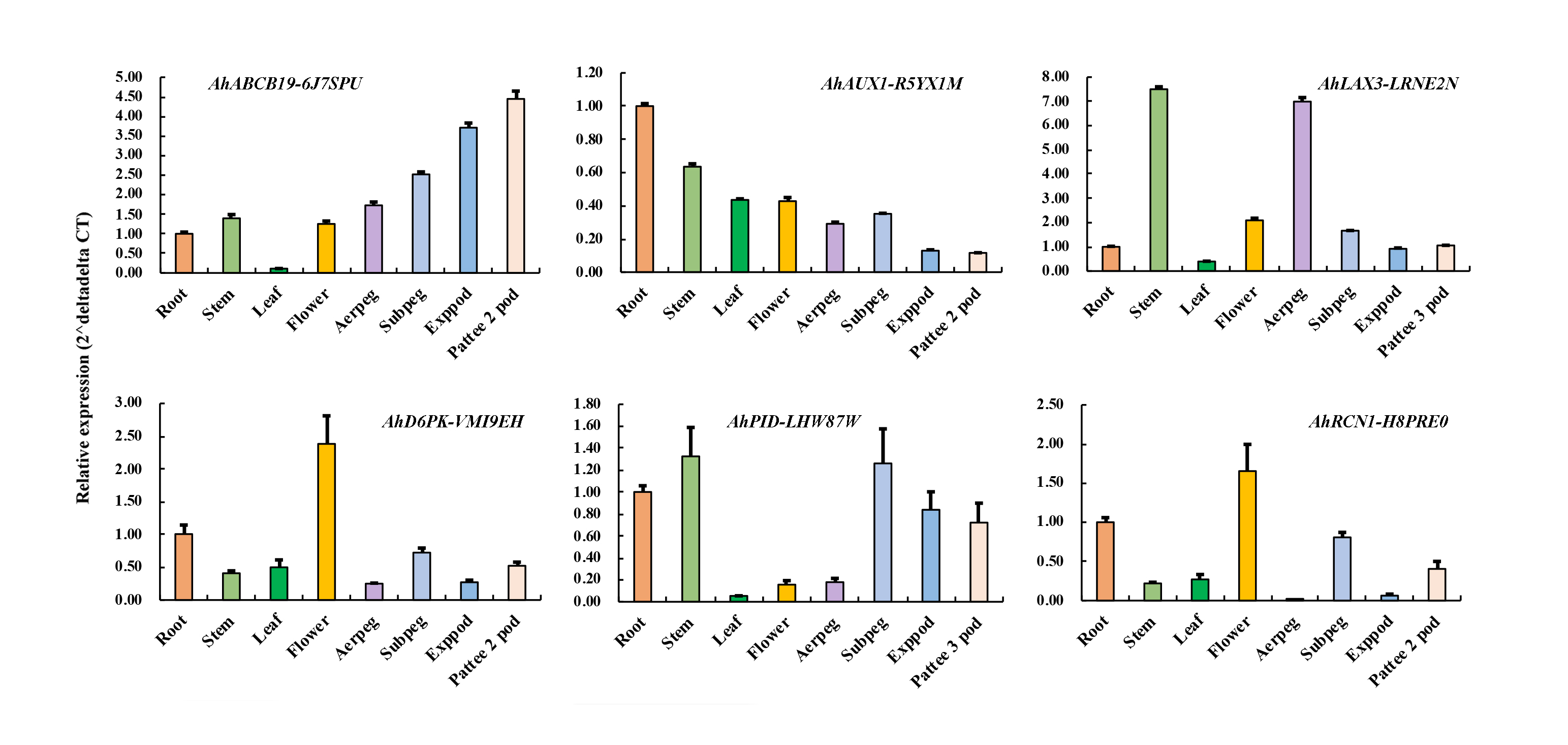
**

**Supplemental Table 1** PIN proteins from Arabidopsis, rice and peanut.

**Supplemental Table 2** Domain of *AhPIN* genes predicted using HMMER with default parameters.

**Supplemental Table 3** Characteristics of cis-acting regulatory elements in the promoter regions of the *AhPIN* genes.

**Supplemental Table 4** The duplication information of *AhPINs*.

**Supplemental Table 5** The Ka/Ks ratios and estimated divergence time between peanut and other species.

**Supplemental Table 6** FPKM value of *AhPIN*s.

**Supplemental Table 7** Raw data of qRT-PCR.

**Supplemental Table 8** Protein interaction analysis of AhPINs proteins.

**Supplemental Table 9** The homologous peanut proteins within the network and FPKM values in the RNA-seq data.

**Supplemental Table 10** Significant Go term of proteins in the interaction network.

**Supplemental Table 11** Primers for qRT-PCR.

**Supplemental Table 12** Primers for constructing the 1300-GFP vector.
